# The influence of the coadministration of the p-glycoprotein modulator elacridar on the pharmacokinetics of lapatinib and its distribution in the brain and cerebrospinal fluid

**DOI:** 10.1007/s10637-019-00806-3

**Published:** 2019-06-08

**Authors:** Agnieszka Karbownik, Katarzyna Sobańska, Włodzimierz Płotek, Tomasz Grabowski, Agnieszka Klupczynska, Szymon Plewa, Edmund Grześkowiak, Edyta Szałek

**Affiliations:** 1 Department of Clinical Pharmacy and Biopharmacy, Poznań University of Medical Sciences, ul. Św. Marii Magdaleny 14, 61-861 Poznań, Poland; 2 Department of Anaesthesiology and Intensive Therapy Teaching, Poznań University of Medical Sciences, ul. Św. Marii Magdaleny 14, 61-861 Poznań, Poland; 3 Polpharma Biologics, ul. Trzy Lipy 3, 80-172 Gdańsk, Poland; 4 Department of Inorganic and Analytical Chemistry, Poznań University of Medical Sciences, ul. Grunwaldzka 6, 60-780 Poznań, Poland

**Keywords:** Lapatinib, Elacridar, Blood-brain barrier, Blood-cerebrospinal fluid barrier

## Abstract

*Background* Lapatinib is a small-molecule tyrosine kinase inhibitor of human epidermal receptor 2 (HER2) and EGFR that has currently been approved for the treatment of HER2-positive advanced and metastatic breast cancer (BC). The ATP-binding cassette (ABC) family of transporters includes P-glycoprotein (P-gp; ABCB1) and breast cancer resistance protein (BCRP; ABCG2), which substantially restrict the penetration of drugs, including chemotherapeutics, through the blood-brain barrier and blood-cerebrospinal fluid barrier. The aim of this study was to investigate the effects of elacridar, an ABCB1 and ABCG2 inhibitor, on the brain and cerebrospinal fluid uptake of lapatinib. *Methods* Rats were divided into two groups: one group received 5 mg/kg elacridar and 100 mg/kg lapatinib (an experimental group), and the other group received 100 mg/kg lapatinib (a control group). Lapatinib concentrations in the blood plasma (BP), cerebrospinal fluid (CSF) and brain tissue (BT) were measured by liquid chromatography coupled with tandem mass spectrometry. *Results* Elacridar significantly increased lapatinib penetration into the CSF and BT (C_max_ increase of 136.4% and 54.7% and AUC_0-∞_ increase of 53.7% and 86.5%, respectively). The C_max_ of lapatinib in BP was similar in both experimental groups (3057.5 vs. 3257.5 ng/mL, respectively). *Conclusion* This study showed that elacridar influenced the pharmacokinetics of lapatinib. The inhibition of ABCB1 and ABCG2 transporters by elacridar substantially enhanced the penetration of lapatinib into the CSF and BT. The blocking of protein transporters could become indispensable in the treatment of patients with breast cancer and brain metastases.

**Electronic supplementary material:**

The online version of this article (10.1007/s10637-019-00806-3) contains supplementary material, which is available to authorized users.

## Introduction

Breast cancer (BC) is the most common malignant cancer diagnosed in women in Europe and the US. Distant metastases are a major threat in the course of the disease [1]. BC is the second most common tumor (following lung cancer) with brain metastases (BM). Approximately 6–16% of patients with BC have metastases within the central nervous system (CNS) [2, 3]. The treatment of BC patients with BM remains a difficult therapeutic problem. The appearance of metastases in the CNS always results in poor prognosis [4–7]. The average survival time of untreated patients is only one month, whereas radiotherapy applied to the CNS extends the survival time to 3–6 months. Even patients with a single CNS lesion who undergo surgery and radiotherapy live only slightly longer, i.e., 10–16 months. The percent of clinically apparent BM is significantly higher (24–48%) in patients with HER2 receptor overexpression [8]. The survival time of these patients is longer than the survival time of patients that are HER2(−). Trastuzumab is a first-line drug used to treat patients with HER2-positive BM. However, due to the high molecular weight of trastuzumab (145,531 Da), its ability to cross the blood-brain barrier (BBB) is limited. Therefore, small-molecule tyrosine kinase inhibitors (TKIs), such as lapatinib, have been the subject of numerous clinical trials to evaluate their effectiveness in women with HER2(+) BM [9, 10].

The results of molecular studies led to the expectation that lapatinib could be successfully applied to BC patients with BM because of its theoretical ability to cross the BBB, as a result of its very low molecular weight (581 Da). Lapatinib was proven to be the first TKI that blocks HER2 receptors and significantly reduces the number of BM [11–13]. Unfortunately, only approximately 2.6–6.0% of patients with BM respond to lapatinib therapy [14]. This minimal therapeutic effect may be caused by the low bioavailability of the drug in the target tissue. This may partly result from the activity of P-glycoprotein (P-gp; ABCB1) and breast cancer resistance protein (BCRP; ABCG2), which are present in the BBB. As these proteins significantly restrict lapatinib penetration into the brain, it is necessary to examine a therapeutic strategy that will overcome this blockade and thus increase the distribution of lapatinib in BM. Therefore, the aim of this study was to assess the influence of elacridar, a third-generation ABCB1 inhibitor, on brain and CSF exposure to lapatinib in rats.

## Materials and methods

### Reagents

Lapatinib (CAS number 231277–92-9), formic acid, methanol, acetonitrile, ammonium formate, and dimethyl sulfoxide (DMSO) were purchased from Sigma-Aldrich (Poznań, Poland). Erlotinib (CAS number 183321–74-6) and elacridar (CAS number 143664–11-3) were purchased from LGC Standards (Łomianki, Poland). Water used in the mobile phase was deionized, distilled and filtered through a Millipore system (Direct Q3, Merck Millipore, Burlington) before use. Lapatinib (Tyverb®_,_ batch number Y68Y) and 0.9% NaCl (batch number 15BO4G61) were purchased from Novartis Polska Sp. z o.o., (Warsaw, Poland) and Baxter Polska Sp. z o.o. (Warsaw, Poland), respectively.

### Animals

This study was conducted on rats, which are a promising model to predict P-gp-based drug-drug interactions at the human BBB [15]. The animals were allowed to acclimate for one week before the beginning of the experiments. Adult male Wistar-strain rats (weight 470–525 g) were used in the study. The animals were maintained under standard breeding conditions with a 12/12 h light-dark cycle (lights on at 6.00 a.m., lights off at 6.00 p.m.) at constant room temperature (23 ± 2 °C), relative humidity (55 ± 10%) and ad libitum access to food and water. The experimental protocol for this study was reviewed and approved by the Local Ethics Committee. To obtain consistent data, the study was based on the required minimum number of animals and observation time. The rats were divided into two groups: one receiving elacridar and lapatinib (I_E + L_) and the other receiving lapatinib (II_L_). Three rats were used at each time point (*n* = 3). In total, the research was conducted on 66 animals (*n* = 33 in the experimental group and n = 33 in the control group). Twenty-two animals were excluded from the research (*n* = 11 from the experimental group and n = 11 from the control group) because their CSF was stained with blood.

### Drug administration and sample collection

Elacridar was freshly dissolved in a vehicle at an initial concentration of 20 mg/mL. This solution was administered intraperitoneally at a dose of 5 mg/kg 30 min prior to lapatinib administration [16]. Lapatinib (100 mg/kg b.w. [17]) was dissolved in 1 mL of 10% DMSO and administered with a gastric probe directly into the rats’ stomach. A volume of 80 μL of blood was collected from each rat by cutting off a piece of its tail. The blood samples were collected into heparinized test tubes at the following time points: before treatment (time 0), 5 min, 30, min, and 1, 2, 3, 4, 6, 8, 12, and 24 h after administration of the drugs, and they were immediately centrifuged at 2880 *g* for 10 min at 4 °C. The anaesthetized rats (anaesthetized by intramuscular administration of 50 mg/kg ketamine and 10 mg/kg xylazine) were prepared for cisterna magna puncture. The hair over the puncture was removed and the skin was disinfected with alcohol. The animals were placed in a recumbent position with an elevated rear part of the body. The animals’ heads were slightly flexed and immobilized with the operator’s left hand. The space between the occiput rostrally and the atlas (C1) of the spinal column caudally was gently palpated with the left thumb. A 0.7 × 20 G needle with polyethylene tubing (Terumo Poland, Warsaw, Poland) was gently inserted at an approximate angle of 80–90° through the skin with the operator’s right hand into the midsagittal region. The moment that dura mater perforation was perceptible, a small amount of CSF was found in the needle tubing. To sample additional CSF, a 1 mL insulin syringe (Becton Dickinson, Warsaw, Poland) was used to collect 80–120 μL of CSF by very gentle aspiration. Only clear CSF was collected for further analysis. At each time point, one animal had to be rejected from the analysis because the collected CSF was stained with blood. The animals’ brains were immediately dissected, washed in 0.9% NaCl and divided along the longitudinal axis. One of the brain hemispheres was again rinsed three times with 5 mL of 0.9% NaCl, weighed and homogenized with 0.9% NaCl (4 mL per 1 g of BT) in an Ultra-Turrax homogenizer (Witko, Łódź, Poland). The homogenate was then centrifuged at 4500 *g* for 10 min. All samples of the rat BP, CSF, and BT homogenate supernatant were stored at −80 °C until analysis.

### Lapatinib measurements

The concentrations of lapatinib in rat BP, CSF and BT homogenates were measured using a validated HPLC method coupled with tandem mass spectrometry (HPLC-MS/MS). The chromatographic analysis was conducted using a 1260 Infinity system (Agilent Technologies, Santa Clara, USA) equipped with a Kinetex® C18 column (50 × 4.6 mm, 2.6 μm, Phenomenex, Torrance, USA), which was maintained at 35 °C. The mobile phase consisted of 0.1% formic acid (*v*/*v*) in water with 5 mM ammonium formate (solvent A) and acetonitrile with 10% phase A (*v*/*v*, solvent B). The analyte and internal standard (IS, erlotinib) were eluted using a gradient run of 10 min as follows: 95% A at 0–2 min; 95%–5% A at 2–4 min; 5% A at 4–5 min; 5%–95% A at 5–6 min; 95% A at 6–10 min. The flow rate and injection volume were set at 0.7 mL/min and 10 μL, respectively. The needle was washed for 30 s in a mixture of acetonitrile, methanol, isopropanol, water, and formic acid (50:20:15:15:0.25, *v*/*v*/*v*/*v*/*v*). A 4000 QTRAP triple quadrupole mass spectrometer (Sciex, Framingham, USA) with an electrospray ionization (ESI) source was operated in multiple reaction monitoring mode. The ESI source parameters were as follows: ion spray voltage - 4000.0 V; nitrogen curtain gas - 45.0 psi; temperature - 500 °C; ion source gas 1–50.0 psi; ion source gas 2–40.0 psi. Positive ionization mode and a dwell time of 160.0 ms were used for all transitions monitored. The following precursor-ion-to-product-ion transitions were measured: *m/z* 581.1 → 365.1 (collision energy, CE 55 eV), *m/z* 581.1 → 350.1 for lapatinib (CE 55 eV) and *m/z* 394.1 → 278.1 for IS (CE 46 eV).

Rat BP (20 μL) was mixed with 50 μL of the IS solution (100 ng/mL) and 930 μL of methanol and then vortexed for 30 s (total dilution factor of 50). After centrifugation at 14,300 *g* (2 min), the supernatant was transferred into an HPLC vial and injected onto the HPLC column.

CSF (20 μL) was added to 10 μL of the IS solution (100 ng/mL) and 170 μL of methanol, vortexed for 30 s, and then centrifuged at 14,300 *g* for 2 min. Next, the supernatant was analyzed by means of the HPLC-MS/MS system. A total dilution factor of 10 was measured.

Rat BT homogenates were diluted with ultrapure water (1:1, *m*/*m*). Then, an aliquot (40 μL) was mixed with 50 μL of the IS solution (100 ng/mL) and 910 μL of methanol, which yielded a total dilution factor of 50. The mixture was vortexed rigorously for 30 s and centrifuged at 14,300 *g* for 2 min. The resulting supernatant was injected onto the HPLC-MS/MS system.

Data acquisition and processing were controlled with Analyst 1.5.2 software (Sciex, Framingham, USA). The calibration curves were prepared within a range of 0.25–150.0 ng/mL with a correlation coefficient r > 0.99. The lower limit of quantification (LLOQ) was determined at 0.25 ng/mL with acceptable precision and accuracy and S/*N* > 10. The accuracy, determined as %bias, was ≤13% across three quality control (QC) levels and < 20% for the LLOQ. The intra- and inter-run precision of the assay (coefficient of variation) was within 15% for the QC samples and below 20% for the LLOQ. Stability experiments with low and high QC samples showed that lapatinib was stable for three freeze-thaw cycles at −80 °C (deviation <5%) and for three months of storage at −80 °C (deviation <15%). Moreover, the postpreparative stability of lapatinib in both BP samples and standard solutions showed no significant change in the concentration (<15%) after 24 h and 48 h of storage in the autosampler at 4 °C.

### Pharmacokinetic analysis

Noncompartmental analysis and the sparse sampling technique in the Phoenix® WinNonlin® 8.0 software (Certara L.P.) were used to calculate the pharmacokinetic parameters on the basis of lapatinib concentration in the rat BP, CSF, and BT. The maximum concentration (C_max_) and the time to reach the maximum plasma concentration (t_max_) were calculated directly from the concentration-time data. The elimination rate constant (k_el_) was estimated from the slope of the terminal linear segment of the log mean concentration-time plot using the automatic best-fitting option in WinNonlin 8.0. The elimination half-life (t_0.5_) was calculated from the ln 2/k_el_. The area under the concentration-time curve from zero to the time of the last concentration measured (AUC_0-t_) was calculated by the linear trapezoidal rule. The residual area under the curve (AUC_res_) was estimated by extrapolation from the last concentration measured (C_last_) to infinity using the C_last_/k_el_ ratio. The area under the curve from zero to infinity (AUC_0-∞_) was computed as the sum of AUC_0-t_ and AUC_res_. The total concentrations in BT were corrected for the analytes present in residual brain blood using the method described by Fridén et al. [18]: C_b,corr_ = [C_b_ - (f_u,p_·V_w_·C_p_ + (1-f_u,p_)·V_protein_·C_p_)]/[1- V_w_], where C_b,corr_ denotes the total concentration of the compound in an individual rat brain corrected for the residual blood; C_b_ is the total concentration measured in an individual rat brain; C_p_ is the mean of the total plasma concentration of the analyte observed in the rats of a given sex and age at the same time as in the brain; f_u,p_ is the unbound fraction of the compound in plasma; and V_w_ and V_protein_ are the apparent brain vascular spaces of plasma water (10.3 mL/g) and plasma proteins (8.0 mL/g), respectively. The tissue-to-plasma partition coefficient (K_p_) was calculated as AUC_0-∞-tissue_/AUC_0-∞-plasma_. The tissue uptake efficiency and selectivity were assessed with K_p_, and the drug targeting index (DTI) was calculated as K_p I(E + L)_/K_pII(L)_ [19]. Additionally, the hysteresis-like plots correlating the plasma concentration with the tissue concentration and the corresponding calculated r^2^ values were analyzed.

## Results

### Concentration-time profiles of lapatinib in BP, CSF, and BT

**Fig. 1:**
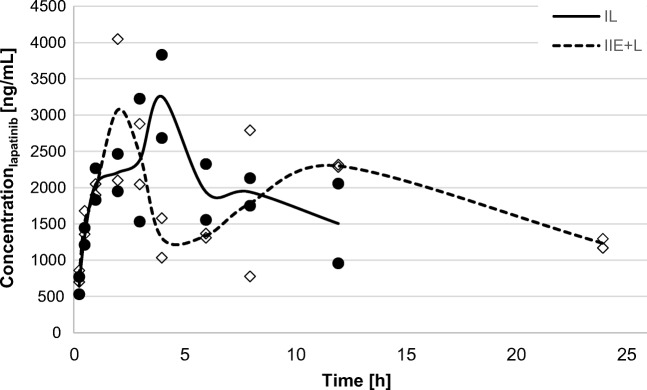
The blood plasma (BP) concentration-time profiles of lapatinib after a single oral dose (100 mg/kg) in the II_L_ (*n* = 2) and I_E + L_ groups (n = 2)

**Fig. 2:**
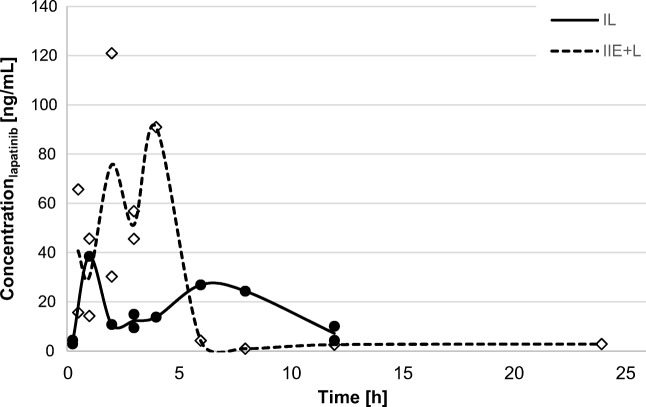
The cerebrospinal fluid (CSF) concentration-time profiles of lapatinib after a single oral dose (100 mg/kg) in the II_L_ and I_E + L_ groups

**Fig. 3:**
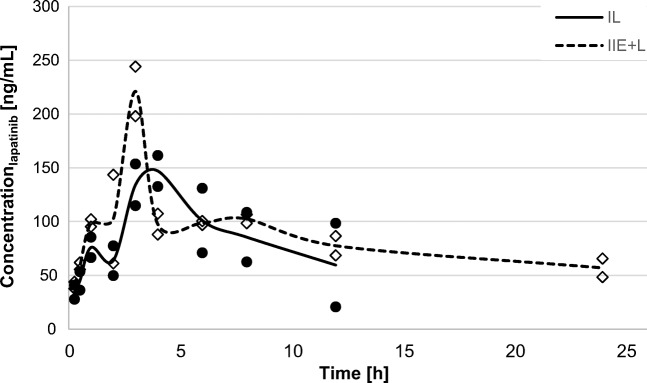
The brain tissue (BT) concentration-time profiles of lapatinib after a single oral dose (100 mg/kg) in the II_L_ (n = 2) and I_E + L_ groups (n = 2)

Figures 1, 2 and 3 show time-dependent changes in the mean concentration of lapatinib in the BP, CSF and BT, respectively. After 24 h, the concentration of lapatinib in each matrix sample in the II_L_ group was undetectable. The levels of lapatinib were below the LLOQ in 20 CSF samples.

### Plasma pharmacokinetic parameters of lapatinib

The pharmacokinetic profile of lapatinib in BP differed between the two groups. The lapatinib plasma AUC_0-t_ and AUC_0-∞_ where 78.7% and 155.6% higher in the I_E + L_ when compared to the II_L_ group (Table 1). The C_max_ of lapatinib was greater by 6.3% and occurred earlier in the I_E + L_ group (2.50 vs. 4.00 h). Moreover, the t_0.5_ of lapatinib was 5.2-fold prolonged when the drug was coadministered with elacridar.

**Table 1:** The noncompartmental plasma pharmacokinetic parameters of lapatinib after oral administration of 100 mg/kg (II_L_) or coadministration with 5 mg/kg of elacridar (I_E + L_)

Pharmacokinetic parameters	I_L + E_	II_L_
Blood plasma (BP)^a^		
C_max_ (ng/mL)	3462.50 ± 823.78	3257.50 ± 809.64
t_max_ (h)	2.50 ± 0.71	4.00 ± 0.00
k_el_ (h^−1^)	0.02 ± 0.01	0.13 ± 0.03
t_0.5_ (h)	28.77 ± 6.00	5.58 ± 1.28
AUC_0-t_ (ng × h/mL)	43,556.06 ± 3905.44	24,377.19 ± 125.07
AUC_0-∞_ (ng × h/mL)	95,087.03 ± 10,426.84	37,197.82 ± 9150.67
AUMC_0-t_ (ng × h^2^/mL)	480,290.03 ± 21,589.07	138,877.66 ± 11,299.79
AUMC_0-∞_ (ng × h^2^/mL)	3,917,566.05 ± 1,363,043.99	404,148.02 ± 215,793.89
MRT_0-t_ (h)	11.05 ± 0.50	5.70 ± 0.43
Cerebrospinal fluid (CSF)		
C_max_ (ng/mL)	91.00	38.50
t_max_ (h)	4.0	1.0
k_el_ (h^−1^)	0.09	0.23
t_0.5_ (h)	7.69	3.00
AUC_0-t_ (ng × h/mL)	355.83	220.76
AUC_0-∞_ (ng × h/mL)	387.33	252.03
AUMC_0-t_ (ng × h^2^/mL)	1620.02	1257.21
AUMC_0-∞_ (ng × h^2^/mL)	2725.51	1767.90
MRT_0-t_ (h)	4.55	5.70
K_p_	0.004	0.007
Brain tissue (BT)^a^		
C_max_ (ng/mL)	197.67 ± 39.01	127.76 ± 16.38
k_el_ (h^−1^)	0.05 ± 0.02	0.25 ± 0.24
t_0.5_ (h)	13.01 ± 3.99	5.29 ± 5.11
t_max_ (h)	3.00 ± 0.00	4.50 ± 2.12
AUC_0-t_ (ng × h/mL)	1610.22 ± 130.95	836.10 ± 21.20
AUC_0-∞_ (ng × h/mL)	2520.56 ± 610.81	1351.27 ± 712.37
MRT_0-t_ (h)	9.92 ± 1.04	5.73 ± 0.06
AUMC_0-t_ (ng × h^2^/mL)	16,040.19 ± 2981.02	4792.63 ± 173.92
AUMC_0-t_ (ng × h^2^/mL)	57,148.33 ± 29,162.29	17,455.77 ± 17,542.73
K_p_	0.027	0.036

C_max_ – maximum concentration; t_max_ – time to C_max_; k_el_ – elimination rate constant; AUC_0-t_ – area under the plasma concentration-time curve from zero to the time of the last measurable concentration; AUC_0-∞_ – area under the plasma concentration-time curve from zero to infinity; t_0.5_ – elimination half-life; MRT_0-t_ – mean residence time; AUMC_0-t_ – area under the first moment curve from zero to the time of the last measurable concentration; AUMC_0-∞_ – area under the first moment curve from zero to infinity; K_p_ – tissue-to-plasma partition coefficient;

^a^ – arithmetic means ± standard deviations

### Tissue uptake

The effect of elacridar on lapatinib tissue distribution was evaluated in the CSF and BT. The C_max_ of lapatinib in the CSF was 2.4-fold increased in the I_E + L_ group when compared to the control group (I_L_) (Table 1). Similarly, 1.55-fold increase of lapatinib C_max_ in the I_E + L_ group was observed in BT. The AUC_0-t_ and AUC_0-∞_ of lapatinib in the CSF were elevated by 61.2% and by 53.7% in the I_E + L_ group. Likewise, the exposure to lapatinib in BT was higher in the I_E + L_ group than in the II_L_ group (AUC_0-t_ and AUC_0-∞_ increased by 92.6% and 86.5%, respectively). The elimination of lapatinib was additionally extended in the presence of elacridar, as the lapatinib t_0.5_ in the CSF and BT was 2.6 times and 2.5 times longer, respectively.

### Tissue uptake efficiency

**Fig. 4:**
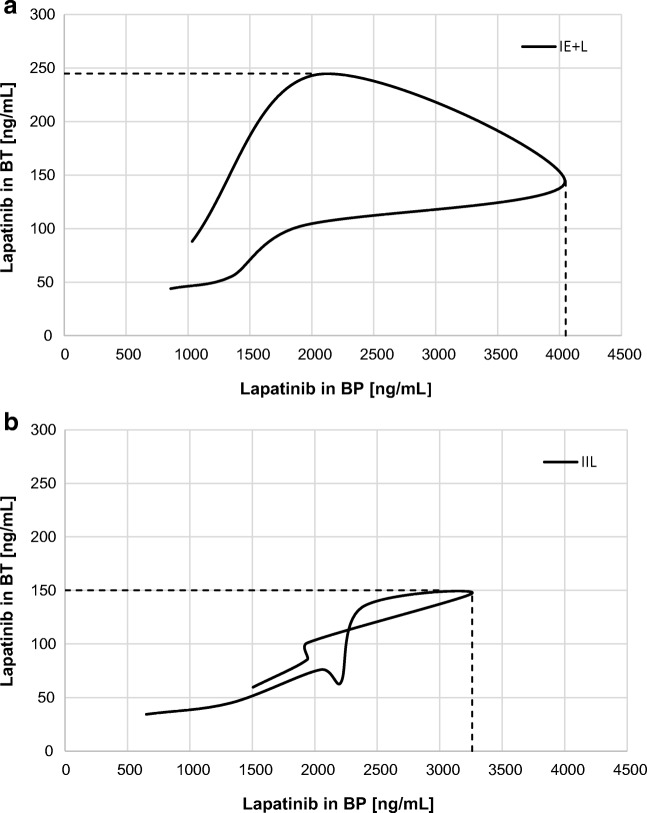
The comparative hysteresis-like plots of the lapatinib concentration in brain tissue (BT) versus blood plasma (BP) for the I_E + L_ group, r^2^ = 0.2109 (**a**) and for the II_L_ group, r^2^ = 0.7751 (**b**)

**Fig. 5:**
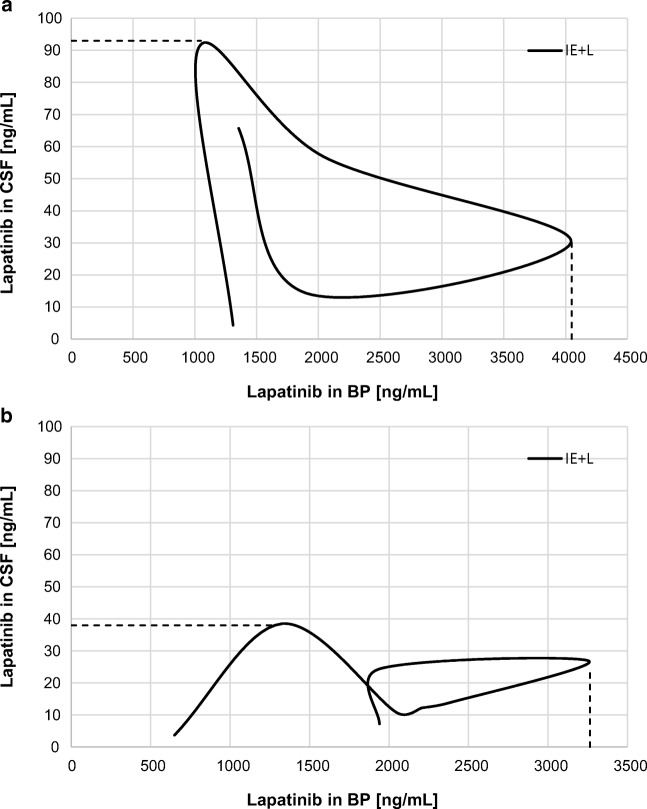
The comparative hysteresis-like plots of the lapatinib concentration in the cerebrospinal fluid (CSF) versus blood plasma (BP) for the I_E_ + _L_ group, r^2^ = 0.1665 (**a**) and for the II_L_ group, r^2^ = 0.0183 (**b**)

The uptake efficiency of lapatinib in the CSF and BT was higher in the II_L_ group than in the I_E + L_ group. The lapatinib K_p_ in the I_L + E_ group was decreased by 42.9% in the CSF and by 25% in the BT when compared to the control group (Table 1). This effect could also be seen in the hysteresis-like plots (Fig. 5), where r^2^ increased slightly in the CSF from 0.0183 in the control group (I_L_) to 0.1665 in the I_E + L_ group. However, the r^2^ value decreased in the BT (0.2109 in the I_E + L_ group vs. 0.7751 in the I_L_ group) (Fig. 4). The BT data above do not allow us to draw conclusions about drug penetration at different doses and concentrations of lapatinib (Fig. 5).

## Discussion

Few anticancer drugs, including cytotoxic and molecularly targeted agents (e.g., TKIs), are sufficiently effective for the treatment of brain tumors. Although the response rate of targeted drugs currently appears to be higher than that observed with conventional chemotherapy in certain subtypes of molecular metastases to BT, it is still insufficient to ensure desirable treatment results. The limited therapeutic potential of available agents against tumors located in BT mainly results from their scarce distribution throughout the BBB. The BBB is characterized by the presence of membrane transporters (e.g., P-gp and BCRP), which are responsible for the efflux of many drugs, including TKIs [20]. However, research has proven that TKIs, such as lapatinib, erlotinib, gefitinib, afatinib, crizotinib, and alectinib, in combination with cytotoxic drugs or alone, are potent in reducing the development of CNS tumors [21–26]. Lapatinib is a substrate for P-gp and BCRP, which are abundantly expressed in the intestine, liver, kidney and BBB [27]. This drug was also identified as an inhibitor of P-gp, BCRP, and OATP (organic anion transporting polypeptide) [28]. Therefore, the knowledge of interactions between lapatinib and these transporters enables an assessment of the possibility of drug penetration into crucial targeted tissues. The inhibition of P-gp-mediated drug efflux has already been recognized as an attractive target for therapeutic intervention to treat multidrug resistant cancers. Elacridar is a potent inhibitor of the proteins ABCB1 and ABCG2 [21, 29]. The inhibited transporters affect the absorption of TKIs in the intestine, their hepatic and renal excretion and penetration through the BBB. The expression levels and activity of P-gp and BCRP are strongly associated with the limited distribution of most TKIs to the BT [22]. Research has shown that brain accumulation of axitinib, cediranib and crizotinib depends mainly on ABCB1 activity, whereas the brain disposition of sorafenib is predominantly influenced by the ABCG2 protein [23].

### Plasma pharmacokinetic parameters of lapatinib

In our study, the coadministration of elacridar substantially change the exposition of lapatinib (Fig. 1). The C_max_ of lapatinib was 6.3% higher in the I_E + L_ group than in the II_L_ group. This may be explained by the fact that systemic exposure to lapatinib is not dependent on gastrointestinal P-gp and BCRP activity. Polli et al. proved that plasma AUC, C_max_ and t_max_ values of lapatinib in rats pretreated with elacridar were similar to the values observed in vehicle-treated animals [28]. However, in our study the AUC_0-t_ and AUC_**0-∞**_ of lapatinib were 78.7% and 155.6% greater than in the II_L_ group (Table 1). Elacridar is highly bound to plasma proteins (in rats: 99.0 ± 0.6% [30], 98.1 ± 1.7 [16]), therefore it could have displaced lapatinib from its plasma protein binding, increasing its free fraction in blood. This could also have resulted in prolonged elimination of lapatinib in the presence of elacridar. The higher exposure to lapatinib might be at least in part a result of the fact that pharmacokinetic calculations were made in I_E + L_ group up to 24 h. In the II_L_ group last measurable point was 12 h, therefore AUC values in this group were underestimated when compared to the I_E + L_ group.

### Tissue uptake and efficiency in cerebrospinal fluid

Elacridar increased the lapatinib C_max_ and AUC_0-∞_ in the CSF 2.4-fold and 1.5-fold, respectively (Table 1). The lapatinib C_max,CSF_/C_max,BP_ ratio proved to be higher in the I_E + L_ group than in the II_L_ group (0.026 vs. 0.012) and the C_max_,_CSF,IE + L_/C_max_,_CSF,IIL_ ratio amounted to 2.4. Gorgi [26] presented a case report of 2 patients treated with lapatinib in combination with capecitabine due to BC with BM (HER2+). Five hours after the oral administration of 1250 mg of lapatinib, the drug concentrations in the BP were 1515 and 3472 ng/mL in the one and another patient, whereas the drug concentration in CSF amounted to 1.3 and 4.5 ng/mL, respectively. The lapatinib C_CSF,5h_/C_BP,5h_ ratios for these two patients were 0.00086 and 0.0013. The authors concluded that the low concentration of lapatinib in the CSF and low ratios may have resulted from the poor solubility of lapatinib in water and low concentrations of proteins in the CSF. In addition, the experiment showed that the concentration of lapatinib in the CSF is not be a reliable indicator of CNS drug penetration.

### Tissue uptake and efficiency in brain tissue

Elacridar significantly increased the lapatinib C_max_ and AUC_0-∞_ in BT 1.5 times and 1.9 times, respectively (Table 1). After the administration of elacridar with another TKI, sunitinib, the penetration of the TKI to the brain was enhanced [19]. The brain-to-plasma ratio of sunitinib in the WT mice after concomitant administration of elacridar was comparable to the brain-to-plasma ratio observed in the knockout mice *(mdr1a/b*^*(−/−)*^*;bcrp1*^*(−/−)*^*).* These data allowed us to conclude that sunitinib penetration to the brain may be increased by the administration of a dual P-gp/BCRP inhibitor. These data can be used to plan clinical trials on patients with brain tumors, e.g., with *glioblastoma multiforme*. Lagas et al. [24] observed that *abcb1a/lb;abcg2*^*(−/−)*^ mice had the most pronounced increase in dasatinib concentration in the brain, which was 13.2 times greater after oral administration and 22.7 times greater after intraperitoneal administration. Moreover, the coadministration of elacridar and dasatinib to WT mice resulted in a similar accumulation of dasatinib in the brain as for *abcb1a/1b,abcg2*^*(−/−)*^ mice. The brain-to-plasma ratio in the WT mice increased 4.4 times with elacridar, but the presence of this P-gp/BCRP inhibitor did not produce similar increase in the P-gp and BCRP knockout mice. Tang et al. [25] showed that *abcb1a/1b*^*(−/−)*^ and *abcb1a/1b;abcg2*^*(−/−)*^ mice experienced approximately 2 times higher BP exposure to low doses of crizotinib (5 mg/kg b.w.) than WT mice. Additionally, the brain accumulation of crizotinib after 24 h was approximately 40 times greater in the WT mice, whereas when the dose was increased ten-fold, the brain accumulation of this TKI in the *abcbla/lb*^*(−/−)*^ mice was approximately 70 times greater than in the WT mice. It is noteworthy that the oral administration of elacridar enhanced the plasma and brain concentrations of crizotinib as well as the brain to plasma concentration ratio in WT mice, which is comparable to that of the *abcbla/1b;abcg2*^*(−/−)*^ mice. The authors concluded that the administration of elacridar with crizotinib could significantly increase the bioavailability and penetration of crizotinib to the BT, and this combination therapy may be used in clinical practice to potentiate crizotinib efficacy in patients with non-small-cell lung cancer and BM.

Oberoi et al. [19] observed that the DTI_BT_ of sunitinib was 3.9 in *mdr1a/b*^*(−/−)*^ mice and 2.1 in *bcrp1*^*(−/−)*^ mice, whereas in mice with both knockout genes, the DTI_BT_ value increased to 48.9. Similarly, Polli et al. [31] showed that after 24-h intravenous administration of lapatinib, the K_p,BT_ value was only 0.04 in WT mice, but it was 40 times greater in *mdr1a/b*^*(−/−)*^*/bcrp*^*(−/−)*^ mice. These results suggested that both P-gp and BCRP synergistically contributed to the penetration of lapatinib in the brain. Blocking P-gp and BCRP activity with elacridar may be a promising option for increasing the efficacy of TKIs, including lapatinib, in the treatment of brain tumors.

Our study was significantly limited by the fact that the unbound drug fraction in the BP, CSF and BT was not measured. Nonetheless, 99% of lapatinib binds with plasma proteins, mainly with albumin and alpha-1 acid glycoprotein. Thus, the unbound fraction is only 1% [32]. We did not use animals with the knockout genotype (*mdr1a/b*^*(−/−)*^*,bcrp1*^*(−/−)*^) to bring the study conditions closer to the physiological state.

## Conclusion

The study proved that elacridar influences the pharmacokinetics of lapatinib. The inhibition of ABCB1 and ABCG2 transporters by elacridar substantially enhanced the penetration of lapatinib into the CSF and BT. Blocking protein transporters is a promising method of increasing the CNS disposition of TKIs and could become an indispensable part of the treatment of patients with BC and brain (micro) metastases behind a functionally intact BBB.

## Electronic supplementary material

ESM 1 (PDF 493 kb)
