# Supplementary material for: The influence of the coadministration of the p-glycoprotein modulator elacridar on the pharmacokinetics of lapatinib and its distribution in the brain and cerebrospinal fluid
Source: Invest New Drugs. 2019 Jun 8;38(3):574–83. doi: 10.1007/s10637-019-00806-3 (PMC7211195; doi:10.1007/s10637-019-00806-3)
Supplement: Supplementary file 1 — (PDF 493 kb) [file 10637_2019_806_MOESM1_ESM.pdf]

Lapatinib *p.o.* 100 mg/kg b.w.

		BP		CSF		BT	
**Rat**	**Time [h]**	$C_{lapatinib}$ ng/ml	S	$C_{lapatinib}$ ng/ml	S	$C_{lapatinibu}$ ng/ml	S
1	0.25	530	**650.0**	4.47	**3.73**	27.7	**34.28**
2	0.25	770		2.98		40.85	
4	0.5	1210	**1327.5**			36.25	**44.88**
5	0.5	1445				53.5	
10	1	1830	**2047.5**	38.5	**38.50**	66.5	**76.00**
11	1	2265				85.5	
14	2	1950	**2207.5**	10.8	**10.80**	49.83	**63.67**
15	2	2465				77.5	
17	3	3225	**2377.5**	9.49	**12.25**	114.83	**134.25**
18	3	1530		15		153.67	
19	4	3830	**3257.5**		**13.80**	132.67	**147.09**
21	4	2685		13.8		161.5	
22	6	1555	**1940.0**		**26.90**	131	**101.00**
24	6	2325		26.9		71	
26	8	2130	**1940.0**	24.4	**24.40**	108.5	**85.50**
27	8	1750				62.5	
28	12	955	**1505.0**	10.1	**7.23**	20.63	**59.57**
29	12	2055		4.36		98.5	

BP - blood plasma
CSF - cerebrospinal fluid
BT - brain tissue
S - medium value

BT homogenate corrected		
BT_{corr} ng/ml	S	body weight [g]
22.47	**27.87**	405
33.26		395
24.03	**31.53**	380
39.02		370
48.15	**55.48**	390
62.82		385
30.05	**41.35**	390
52.65		380
82.46	**110.90**	385
139.35		385
94.19	**114.71**	390
135.24		380
116.18	**81.86**	450
47.54		475
87.46	**66.20**	445
44.94		465
10.91	**44.52**	450
78.14		460

Lapatinib 100 mg/kg + elacridar

		BP		CSF		BT		BT homog
**Rat**	**Time [h]**	$C_{lapatinib}$ ng/ml	S	$C_{lapatinib}$ ng/ml	S	$C_{lapatinibu}$ ng/ml	S	BT_{corr} ng/ml
34	0.25	700	**780.0**	<LOQ		37.6	**40.78**	30.71
36	0.25	860		<LOQ		43.95		35.46
37	0.5	1680	**1517.5**	15.7	**40.7**	62	**58.75**	45.16
38	0.5	1355		65.7		55.5		41.98
40	1	2050	**1975.0**	45.7	**30**	95.25	**98.63**	74.91
41	1	1900		14.3		102		83.29
43	2	2100	**3072.5**	121	**75.65**	61	**102.25**	39.78
44	2	4045		30.3		143.5		102.90
46	3	2880	**2462.5**	45.6	**51.20**	198	**221.00**	170.09
47	3	2045		56.8		244		225.26
52	4	1578	**1305.5**	<LOQ	**91**	107.25	**97.63**	91.94
53	4	1033		91		88		78.17
56	6	1365	**1337.5**	<LOQ	**4.27**	100.5	**98.67**	87.34
57	6	1310		4.27		96.83		84.20
58	8	775	**1782.5**	<LOQ	**1.03**	98.5	**102.50**	91.46
59	8	2790		1.03		106.5		78.57
62	12	2285	**2300.0**	<LOQ	**2.62**	86.5	**77.50**	63.62
63	12	2315		2.62		68.5		45.12
50	24	1295	**1232.5**	2.84	**2.84**	65.5	**56.925**	52.70
51	24	1170				48.35		36.68

enate	
S	body weight [g]
**33.08**	510
	465
**43.57**	470
	495
**79.10**	525
	465
**71.34**	450
	575
**197.67**	485
	480
**85.05**	475
	485
**85.77**	445
	440
**85.02**	495
	490
**54.37**	445
	450
**44.69**	450
	460

lap	1	22.47	0.25	
	1	33.26	0.25	
	1	24.03	0.5	
	1	39.02	0.5	
	1	48.15	1	
	1	62.82	1	
	1	30.05	2	
	1	52.65	2	
	1	82.46	3	
	1	139.35	3	lapatin
	1	94.19	4	
	1	135.24	4	
	1	116.18	6	
	1	47.54	6	
	1	87.46	8	
	1	44.94	8	
	1	10.91	12	
	1	78.14	12	
lap+el	2	30.71	0.25	
	2	35.46	0.25	
	2	45.16	0.5	
	2	41.98	0.5	
	2	74.91	1	
	2	83.29	1	
	2	39.78	2	
	2	102.9	2	
	2	170.09	3	
	2	225.26	3	
	2	91.94	4	
	2	78.17	4	
	2	87.34	6	
	2	84.2	6	
	2	91.46	8	
	2	78.57	8	
	2	63.62	12	
	2	45.12	12	
	2	52.7	24	
	2	36.68	24	

		S	Sampling point [h]
lapatinib	1	27.86703	0.25
	1	31.52647	0.5
	1	55.48222	1
	1	41.35369	2
	1	110.9041	3
	1	114.7143	4
	1	81.86117	6
	1	66.19986	8
	1	44.52208	12
ib+elacridar	2	33.08174	0.25
	2	43.56851	0.5
	2	79.0972	1
	2	71.33803	2
	2	197.6723	3
	2	85.05441	4
	2	85.7722	6
	2	85.01591	8
	2	54.37001	12
	2	44.69056	24

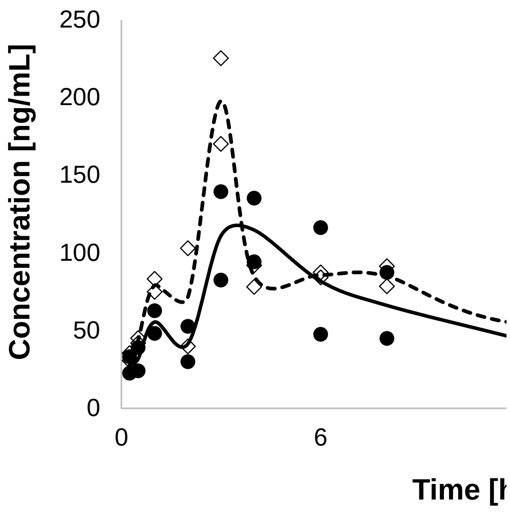

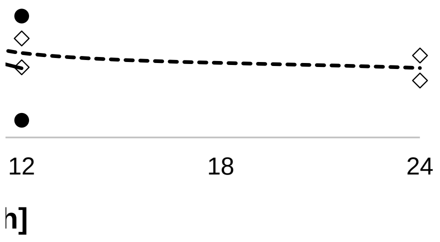
12
18
24
h]

Subject	Ka	SS	WSS	SC	AIC	AUC(0-t)	AUC(0-inf)	AUMC(0-t)	AUMC(0-inf)	MRT(0-t)
	1/h					µg*h/L	µg*h/L	µg*h*h/L	µg*h*h/L	h
1	0.2582	255.77	255.77	58.69	57.9	836.1	1275.27	4792.63	14403.63	5.73
2	0.7828	989.88	989.88	78.19	76.98	1610.22	2882.4	16040.19	84686.49	9.96

MRT(0-inf)	Kel	Y(el)	t1/2kel	Cmax	tmax	Clast	tlast	AUCrest%	AUCrest	Cl	Vss
h	1/h	ng/mL	h	ng/mL	h	ng/mL	h	%	µg*h/L	L/g*h	L/g
11.29	0.1012	149.61	6.85	114.71	4	44.52	12	34.44	439.18	0.025	0.141
29.38	0.0334	94.61	20.77	197.67	3	44.69	24	44.14	1272.18	0.015	0.148
